# A Shared Worldview: Mental Health and Public Health at the Crossroads

**Published:** 2009-12-15

**Authors:** Wayne H. Giles, Janet L. Collins

**Affiliations:** Centers for Disease Control and Prevention; Centers for Disease Control and Prevention, Atlanta, Georgia

The conceptual paradigm underlying public health has expanded dramatically in recent years to include social influences on health, such as poverty, education, housing, justice, and transportation. Despite this more expansive view, the fields of public health and mental health have remained isolated in their respective work. Appreciation for the inseparable relationship between physical and mental health is growing but has largely been insufficient to unite the 2 fields in any meaningful way. The connections between chronic disease, injury, and mental health are particularly striking. For example, the rate of tobacco use among people diagnosed with a mental health condition is approximately twice that of the general population (1). Similarly, injury rates for both intentional (for example, homicide) and unintentional (for example, motor vehicle injuries) injuries are approximately 2 to 6 times higher among people with a mental illness than for the general population (2,3). Although mental health problems appear to precipitate behaviors that compromise health, such as tobacco, alcohol, and substance use, behaviors that enhance health, such as physical activity, can improve mental health status and quality of life (4).

As might be expected, diagnosis of a chronic disease appears to contribute to or exacerbate depression and other mental health conditions. For example, after a heart attack, 1 in 3 patients exhibit depressive symptoms and nearly 1 in 6 are formally diagnosed with depression (5). Results from clinical trials also demonstrate that mental health status plays a role in long-term health outcomes. For example, treating depression can improve the ability of the patient and the doctor to successfully manage ongoing chronic conditions (6).

In the spring of 2008, the Centers for Disease Control and Prevention convened a panel of experts to address opportunities for the mental health and public health communities to work together. The panel included representatives from the Substance Abuse and Mental Health Services Administration, the National Institutes of Mental Health, the American Psychiatric Association, National Association of Chronic Disease Directors, the Carter Center, state mental health directors, and academia. What is clear from the expert panel's recommendations is that the public health and mental health communities must take immediate steps to improve the public's health.

The panel recommended the expansion of the nation's surveillance capacity to address physical and mental health and their intersection. Current surveillance systems, particularly those at the state and local levels, have little ability to measure mental health. Although every state conducts surveillance on chronic conditions, only 17 include measures that simultaneously assess mental health.

Another priority area identified by the panel is the joint training needs of the public health and mental health workforces. Public health practitioners need to better understand the links between physical and mental health outcomes and how to effectively intervene for people with mental health conditions. Likewise, given the high prevalence of physical health conditions among people with mental health conditions, the mental health community must understand and effectively intervene in the prevention, treatment, and control of chronic conditions and injury.

Finally, the panel noted that the public health and mental health communities must do more in the areas of disparities elimination and health equity. Unfortunately, large racial, ethnic, geographic, and socioeconomic disparities exist in mental health outcomes; these disparities are often larger than those seen for physical health conditions (7). Success in improving population-based physical and mental health outcomes requires addressing the root causes of disparities, including poverty, education, employment, health care, and housing.

During the past 10 years, the treatment of many mental health conditions has moved from specialty centers into primary care (7). This change in practice has increased the potential for more integrated care. The links between mental health, public health, and related community support, however, remain disjointed. Community support relevant to both physical and mental health includes appropriate referrals and access to high-quality primary and mental health care, including cessation support for tobacco, alcohol, and other substance use; livable wages and safe housing; free, safe, and attractive places for physical activity; and access to food that is healthy and affordable. Unfortunately, too few communities, especially those in low-income areas, offer comprehensive policy and environmental supports for health.

We commend the panel on its work to establish a vision and integrate health promotion. Movement along this path holds promise for simultaneously improving the physical and mental health of the nation.
