# Erratum, Vol. 7: Issue 1

**Published:** 2010-02-15

In the article "A Shared Worldview: Mental Health and Public Health at the Crossroads," references 6 and 7 were switched in the reference list. The correction was made to our Web site on December 21, 2009, and appears online at http://www.cdc.gov/pcd/issues/2010/jan/09_0181.htm. We regret any confusion or inconvenience this error may have caused.

